# The Relationship between Visual-Evoked Potential and Optic Coherence Tomography and Clinical Findings in Parkinson Patients

**DOI:** 10.1155/2023/7739944

**Published:** 2023-02-23

**Authors:** Zeynep Tuncer, Gamze Dereli Can, Hava Dönmez Keklikoğlu, Fatma Ayşen Eren, Fatma Yülek, Orhan Deniz

**Affiliations:** ^1^Sakarya Adatıp Hospital, Neurology and Pain Clinic, Sakarya, Turkey; ^2^Department of Ophthalmology, Bursa City Hospital, Bursa, Turkey; ^3^Department of Neurology, Ankara Medical Park Hospital, Ankara, Turkey; ^4^Department of Neurology and Pain Management, Kanuni Sultan Süleyman Research and Training Hospital, Istanbul, Turkey; ^5^Department of Ophthalmology, Ankara Yıldırım Beyazıt University Faculty of Medicine, Ankara, Turkey; ^6^Department of Neurology, Ankara Yıldırım Beyazıt University Faculty of Medicine, Ankara, Turkey

## Abstract

**Background:**

In Parkinson's disease (PD), dopamine deficiency is present not only in the nigrostriatal pathway but also in the retinal and visual pathways. Optic coherence tomography (OCT) can be used as morphological evidence of visual influence from early nonmotor symptoms. The aim of this study was to investigate the relationship of OCT and visual evoked potentials (VEPs) of eyes with the severity of clinical findings and ocular findings in PD.

**Methods:**

A group of 42 patients diagnosed with idiopathic PD and a control group of 29 people between the ages of 45–85 were included in our study. VEP was recorded in the patient and control groups. OCT measurement was made with the Optovue spectral-domain device. Foveal thickness and macular volume were measured in the foveal region and in the parafoveal and perifoveal regions in the temporal, superior, nasal, and inferior quadrants. RNFL (retinal nerve fiber layer) was measured in temporal, superior, nasal, and inferior quadrants. Ganglion cell complex (GCC) was evaluated in the superior and inferior quadrants. Using the UPDRS clinical scale, the relationship between measurements and the differences between the control group and the patient group were evaluated.

**Results:**

Among the OCT values in our study, foveal, parafoveal, perifoveal thickness, macular volume, RNFL, and GCC measurements were performed for the right and left eyes, and no difference was found between the patient group and the control group. There was no difference in VEP amplitude and latency values between the patient and control groups. The relationships between UPDRS and modified Hoehn Yahr staging and OCT and VEP measurements in the patient revealed no correlation.

**Conclusions:**

Studies on whether OCT measurements can functionally be a marker or which segments are more valuable for disease progression in patients with PD are needed. Visual dysfunction in PD cannot be attributed only to retinal pathology; however, the retina may provide monitoring of the status of dopaminergic neurodegeneration and axonal loss in PD.

## 1. Introduction

Parkinson's disease (PD) is an idiopathic, progressive disease with four cardinal symptoms such as resting tremor, rigidity, bradykinesia, and postural reflex loss, which primarily stems from nigrostriatal dopaminergic insufficiency [1–5]. The information obtained today on PD, the second most common neurodegenerative disease, points to a multifactorial etiology rather than a single cause for the disease [6]. Not all symptoms occur simultaneously and are not of the same intensity in every patient with PD. Thus, it draws attention that the combinations of symptoms and signs are different in each patient in PD, which is a clinically heterogeneous disease [7–9].

There is increasing evidence that a series of precursor symptoms appear before motor signs develop and that the disease has a prodromal phase of variable length. Most of these forerunners and early symptoms are nonmotor symptoms, contrary to the classical definition. One of the nonmotor features of this period, which is called the premotor period, is the visual disturbances that may be caused by retinal dopamine loss [10–13]. Visual function losses associated with PD, such as impaired color vision, temporal sensitivity, and spatial contrast sensitivity, suggest that they are partially controlled by dopamine and foveal vision is particularly impaired in PD [14–17].

Visual evoked potential (VEP) waveforms are derived from the electroencephalogram (EEG) by signal averaging. VEP is used to measure the functional integrity of visual pathways from the retina to the visual cortex in the brain via the optic nerve [18]. Initially, evidence of visual loss in PD was obtained from measurements of VEP and contrast sensitivity. While many studies with VEP have shown prolonged neural conduction in the visual pathway in PD, the corresponding finding has not been supported in some other studies [19–24].

Optic coherence tomography (OCT) is a noninvasive transpupillary imaging technology that provides high-resolution cross-sectional views of ocular biological structures to evaluate the anatomical layers of the retina [20]. OCT allows detecting of axonal components in the anterior visual pathways. Speaking of retinal layers, the inner retinal layer (IRL) includes the nerve fiber layer (RNFL), the ganglion cell layer (GCL), and the inner plexiform layer (IPL), while the outer retinal layer (ORL) starts from the inner nuclear layer and ends with the retinal pigment epithelium.

This technique has been proposed as a way to monitor PD within the retina. There are a large number of studies using SD-OCT in PD patients; however, conflicting results have been reported [1, 2, 25]. The number of studies examining retinal changes for diagnostic purposes in PD is high in the literature, but the number of studies to determine the relationship between the severity of the disease and retinal changes are few.

The aim of this study was to investigate the relationship between the severity of clinical findings of the disease with retinal functional and structural changes detected by OCT and VEPs in patients with PD.

## 2. Materials and Methods

This study included a 42-person idiopathic PD patient group followed by Ankara Atatürk Training and Research Hospital and a 29-person control group between the ages of 45–85. Patients meet the criteria for the UK Brain bank [26]. The patient group could be under medication or newly diagnosed. All the people in the patient and control groups were informed about the study and their approvals to participate were obtained. The study complied with the Helsinki Declaration requirements and received local ethics committee approval (Ethics Committee Decision Letter No. 20033663-903/126).

All patients with PD took their medications on their evaluation day, being examined during their on-status; past medical history was noted and neurological/neuro-ophthalmological examinations were performed. Pathological data could be more prominent in the off-status, but it was thought that being pathological in the on-status would give more reliable results. The severity of the disease, cognitive functions, activities of daily living, and PD's motor characteristics were evaluated using unified Parkinson's disease rating scale (UPDRS) clinical scale [27]. At the same time, the modified Hoehn Yahr stage was used for the disease severity [28]. The duration and onset side of PD, antiparkinsonian therapy, comorbidities, and other drugs used by the patients were noted.

UPDRS is the most frequently used scale in PD, which is based on clinical examination consisting of four main parts: mental state, daily life activities, motor examination, and treatment complications, and the answers to the questions are given by the patient and the care-giver. This scale was performed by a neurologist.

### 2.1. Visual Acuity, Biomicroscopic, and Intraocular Pressure (IOP) Measurement Records

Visual acuity assessment, biomicroscopic anterior, posterior segment examination, and IOP measured by Goldmann applanation tonometry, and OCT (Carl Zeiss Meditec, Germany) were measured and recorded by an ophthalmologist.

Visual field examination was performed with the confrontation method, and color vision examination was performed with the Ishihara test, which is frequently used as a screening test in clinical practice. Visual acuity examination was performed using near visual acuity charts. Distant visual acuity was examined by an ophthalmologist in patients who could not cooperate or could not look at the reading chart with pinhole due to tremor dominance. Near visual acuity was measured monocularly with one of the standard reading charts using appropriate refractive correction from a distance of 33.5–35 cm. Visual acuity measured with near reading charts was recorded by taking the logMar measurement equivalent. Thus, 20/20 visual acuity is LogMAR 0.00 and 20/200 visual acuity is LogMAR 1.0.

Patients with visual acuity equal to LogMAR0.4 and better than 0.4 and those with IOP less than 22 mmHg and those without color blindness were included in the study. Those with pathologies such as macular degeneration or glaucoma in the posterior pole and retinal degeneration, and those with cataracts that could interfere with OCT and ocular examination were excluded from the study. The examination was performed by an ophthalmologist in terms of cataract examination and other ophthalmological diseases. They were divided into cataract, cortical, corticonuclear cataract, and pseudophakic groups. The conventional ophthalmologic examination included refractive status (RK-F2, Canon), best-corrected visual acuity (Snellen, BCVA), biomicroscopic anterior and posterior segment examinations, and measurement of intraocular pressure (IOP, CT.1P, Topcon).

### 2.2. VEP Records

After VEP recording was explained to the patient and control groups, the keypoint electromyography device was used for measurement. We comply with the international society for clinical electrophysiology of vision (ISCEV) standards for a few differences [29]. Our differences were reversal rate and sweep speed. These changes were used in this way to obtain the most optimal waveform with laboratory conditions and equipment. The person was placed in front of the monitor screen with an eye-screen distance of 100 cm. The ground electrode was connected to the right wrist. The scalp needle electrode was used as an active electrode. Head circumference measurements were made. 10% of the obtained value was taken by measuring between nasion and inion in accordance with the international 10–20 system. The active electrode is placed on the occipital scalp over the visual cortex at Oz with the reference electrode at Fz. During the measurement, a scalp needle electrode was used to lower the impedance and obtain a more objective wave. The monocular recording was performed by covering one eye with an eye pad.

VEP recording was made with a 12 × 16 checkerboard pattern reversal pattern. Pattern-reversal VEPs elicited by checkerboard stimuli with large, 1 degree (°), and small, 0.25° checks. The black and white checks change reverse abruptly, with no overall change in the luminance of the screen. The mean luminance was 50 (cd m^−2^). The contrast between black and white squares was high and Michelson contrast^2^ was 80 (%). Pattern-reversal VEPs were obtained using a reversal rate of 3 reversals per second (rps), 200 averaging settings. The settings of the device were pes frequency 10 Hz, treble frequency 0.1 kHz, and sweeping speed 30 ms/min. The patient was asked to look at the midpoint on the screen as the fixation point. Patients who had difficulty in cooperation were excluded from the study group. Impedance was checked before each procedure and recording was started if it was below 5-kilo ohms. During the registration of the patients, care was taken to ensure that the ambient conditions such as the lighting of the room were the same. After the recording samples were taken from the computer, the electrodes were removed and the procedure was terminated.

In the VEP recorded, N75, P100, and N135 waves were plotted, and latencies and the peak-to-peak amplitude of the *P* wave were measured. Latencies are given in milliseconds (ms) and amplitudes in microvolts (*μ*V). These peaks are designated as negative and positive followed by the typical mean peak time. We used a negative waveform and measurements were taken on this waveform. The standard measure of VEP amplitude is the height of P100 from the preceding N75 peak.

### 2.3. OCT Measurements

OCT measurements for both groups were carried out by the ophthalmologist included in the study. OCT was performed by using the Optovue spectral-domain device. The patient was asked to look fixed at a target point provided by the device during the imaging. And, with the protocol of the device, the foveal thickness was measured in the foveal region, and the parafoveal and perifoveal regions in the temporal, superior, nasal, and inferior quadrants. Macular volume was measured in the foveal region, in the parafoveal and perifoveal regions, and in the temporal, superior, nasal, and inferior quadrants. RNFL was measured in temporal, superior, nasal, and inferior quadrants. Ganglion cell complex (GCC) was evaluated in the superior and inferior quadrants. In mapping, the fovea reflects the areas of 1 mm in the fovea center, 3 mm in the parafovea, and 5 mm in the perifovea.

All analyses were performed on SPSS v22 (SPSS Inc., Chicago, IL, USA). Data are given as mean ± standard deviation or median (minimum-maximum) for continuous variables according to the normality of distribution and as frequency (percentage) for categorical variables. Normally distributed variables were analyzed with the independent samples Student's *t*-test. Non-normally distributed variables were analyzed with the Mann–Whitney *U* test. Frequency distribution for categorical variables and descriptive statistics (mean, standard deviation, minimum, and maximum) for numerical variables were applied. Two-tailed *p*-values of less than 0.05 were considered statistically significant. The correlation between the mean VEP values, OCT measurements, and the UPDRS stages were evaluated by the Spearman correlation test.

## 3. Results

Forty-two patients diagnosed with idiopathic PD were enrolled, 1 patient with inconsistent data was excluded, and 41 patients were included. The right eye was excluded because of maculopathy in the right eye in 1 of 41 patients. Due to technical reasons, the right and left eye RNFL measurement in 1 patient, left perifoveal temporal quadrant thickness and macular volume measurement in 1 patient, and left perifoveal nasal quadrant macular volume and right eye RNFL measurement in 1 patient were not included in the study.

Thirty-one people were enrolled in the control group, however, 29 people were included in the study as 1 patient had macular edema and 1 patient showed glaucomatous changes in OCT measurements. In 1 of the 29 control group participants, left perifoveal nasal quadrant foveal thickness and macular volume, and right eye measurements of 1 patient were not included in the study. Of the 29 people, 21 accepted the VEP measurement.

Visual acuity, color vision visual examination, biomicroscopy, fundus examinations, IOP, OCT, and VEP measurements were performed in all patients. The drugs patients used for PD, the onset side of the disease, the duration of PD, and comorbidities were evaluated. Twenty-one (51.2%) of the patients had right-sided onset, 18 (43.9%) had left-sided onset, and 2 (4.9%) had akinetic rigid form. The mean age of the patient group and the control group was 65.58 ± 9.89 and 63.48 ± 11.23 years, respectively, and there was no significant difference between the mean ages (*p*=0.411). The patient group consisted of 14 women and 27 men and the control group of 17 women and 12 men. Visual acuity LogMAR values are shown in Tables 1 and 2.

**Figure 1:**
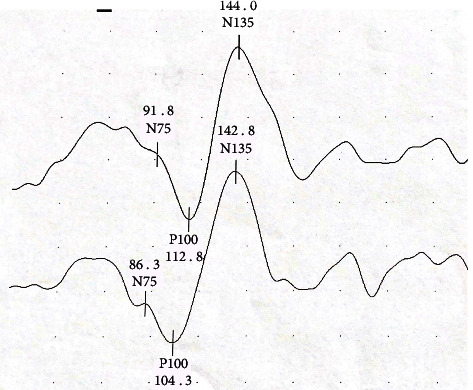
A waveform sample from PD group. VEP amplitude, N75, N135, and P100 latencies for the right and left eyes were measured. The upper waveform was obtained from the right eye and the lower waveform was obtained from the left eye. (Latencies are given in milliseconds (ms) and amplitudes in microvolts (*μ*V). Between two points in the vertical direction represent 2 *μ*V, peak-to-peak amplitude of the *P* wave are 7.66 *μ*V on the upper wave and 7.33 *μ*V on the lower wave, respectively).

**Figure 2:**
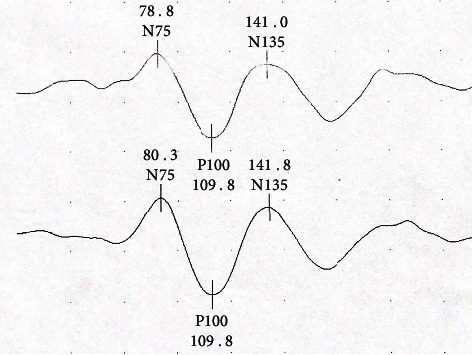
A waveform sample from control group. VEP amplitude, N75, N135, and P100 latencies for the right and left eyes were measured. The upper waveform was obtained from the right eye and the lower waveform was obtained from the left eye. (Latencies are given in milliseconds (ms) and amplitudes in microvolts (*μ*V). Between two points in the vertical direction represents 5 *μ*V. Peak-to-peak amplitude of the *P* wave are 7.85 *μ*V on the upper wave and 9.28 *μ*V on the lower wave, respectively).

In the patient group, VEP amplitude, N75, N135, and P100 latencies, as well as all OCT measurements, foveal parafoveal perifoveal thickness, macular volume, RNFL, and GCC data were evaluated in terms of normal distribution. VEP waveforms from the patient group and control group were shown in Figures 1 and 2, respectively. VEP Latencies are given in milliseconds (ms) and amplitudes in microvolts (*μ*V). In Figure 1, from the patient group; N75, N135, and P100 latencies for the right eye are 91.8 ms, 112.8 ms, and 144 ms and for the left eye are 86.3 ms, 104.3 ms, and 142.8 ms, respectively. VEP amplitude for the right eye is 7.66 *μ*V and for the left eye is 7.33 *μ*V. In Figure 2, from the control group; N75, N135, and P100 latencies for the right eye are 78.8 ms, 109.8 ms, and 141 ms and for the left eye are 80.3 ms, 109.8 ms, and 141.8 ms, respectively. VEP amplitude for the right eye is 7.85 *μ*V and for the left eye is 9.28 *μ*V.

**Figure 3:**
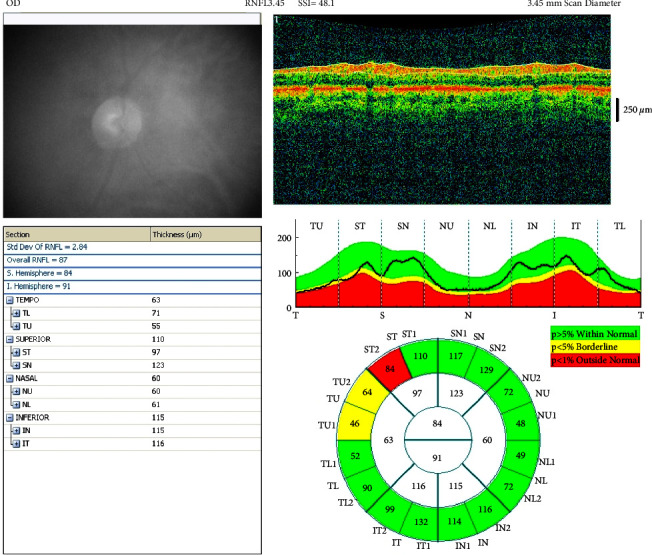
OCT measurement values and image of a patient from the patient group with right RNFL temporal quadrant thinning.

**Figure 4:**
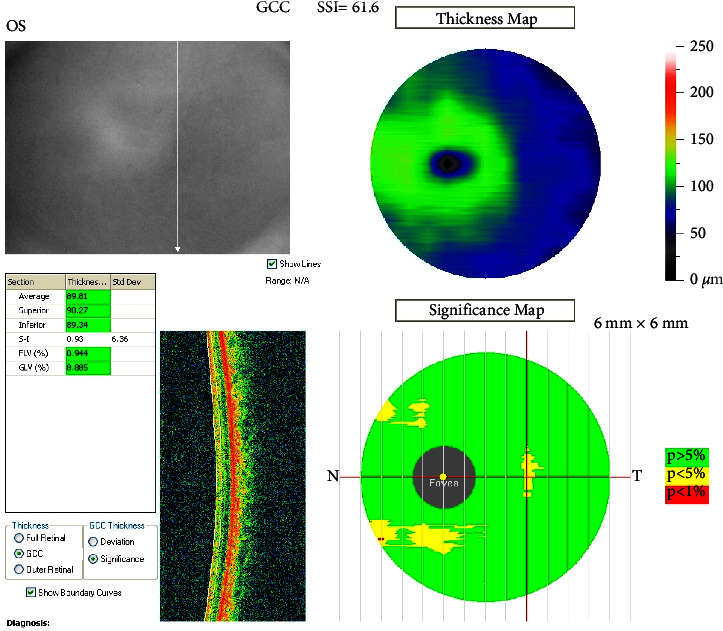
Normal GCC measurement and OCT image of a patient from the patient group.

OCT measurement samples from the patient group are shown in Figures 3 and 4. In Figure 3, the OCT measurement values and image of a patient from the patient group with right RNFL temporal quadrant thinning is presented, while Figure 4 shows the normal GCC measurement and OCT image of a patient from the patient group.

The student's *t*-test was used to measure 40 Right eyes and 41 left eyes in the patient group and 21 right and left eye measurements in the control group. There was no statistically significant difference in VEP latencies and VEP amplitudes between groups. Since PD can have asymmetric onset with clinically heterogeneous symptoms, both the right eye and the left eyes were included in the measurement. Both eyes were evaluated. Comparison of OCT and VEP data in the patient and control groups are shown in Table 3. No significant difference was observed.

The average, minimum, and maximum values of VEP measurements and UPDRS of the patient group are shown in Table 4. Values of 41 patients were compared for VEP results. No significant difference was found between the right and left VEP values of the patient group (Table 5). No significant correlation was identified between the mean VEP values and the UPDRS stages (Table 6).

The distribution of macular volume among the OCT measurements was examined in 4 parafoveal perifoveal quadrants and fovea. Thirty-six eyes were included. No normal distribution was observed. No significant correlation was found between UPDRS scores and macular volume measurements.

## 4. Discussion

In our study, foveal, parafoveal, perifoveal thickness, macular volume, RNFL, GCC measurements, and VEP were measured in the right and left eyes, and no difference was found between the patient group and the control group. Also, we could not find any correlation between UPDRS and VEP and OCT measurements between the patient group and the control group. There are so many different results of other studies about ocular findings in PD but few studies for comparing these ocular findings with the degree of severity of PD.

The highest concentration of receptors in the retina provides neural output to subsequent neural elements and especially RGCs [30]. The structural and functional changes of the retina in PD raise many questions. First, if we consider that the prevalence of Parkinson's disease increases with age, the retinal dysfunction that will occur must be found after age-related ones are revealed. The question, is whether the dopaminergic deficiency causes dysfunction at the retinal level or the lateral geniculate nucleus (LGN) or the visual cortex, or all also come to the mind. Although the answers have been explored for 40 years, some questions remain unanswered [31]. Progressive retinal dopaminergic deficiency leads to the loss of retinal amacrine cells that provide input to RGCs [20, 32, 33]. Since high vision centers, LGN, and visual cortex also contain dopaminergic neurons, they can also be easily affected in PD.

While T-VEP (low variability fast transient VEP) is superior for spatial frequency measurements, S-VEP is superior for temporal frequency measurements [19, 34–36]. We used T-VEP 12 × 16 checkerboard pattern in our measurements and evaluated foveal vision. The foveal region is the region where the cone and ganglion cells are the most concentrated. We preferred T-VEP because of the presence of foveal visual impairment and reflection of ganglion cell function in PD. Neural production sites in VEP formation are not clear. The most valuable and sensitive measurement is the P100 latency, and the difference between the two eyes is also more sensitive. P100 is not just a cortical response. The optic nerve, geniculate body, cortex, and thalamus have quite mixed effects in signaling through the visual system. Axonal loss is expected to change amplitude without creating latency change. There is a more pronounced attenuation of P 100 amplitude than latency with aging [37]. Prolonged VEP has also been described in PD, which is associated with neurotransmitter deficiency rather than a demyelinating disease [17, 38]. It was observed that VEP latencies returned to normal with the application of dopamine precursors [32]. Findings support that VEP latency changes may occur due to synaptic dysfunction as well as impaired conduction [38, 39].

In a study, VEP amplitude and latency were found not to be associated with the asymmetric side of the disease and any clinical features except the bradykinesia score. Due to the positive correlation between bradykinesia score and VEP amplitude, D2 receptors have been suggested to possibly be more common in the retina [23]. However, no correlation was found between VEP and the side, duration, the severity of the disease, and the duration of treatment ın another study [40]. In our study, no difference was found in VEP amplitude and latencies between the patient and control groups. However, at this point, the number of VEP measurements in the patient and control group included in the study should also be taken into account. Forty-one right and left eyes from the patient group and 21 from the control group were included in the present study.

Various VEP abnormalities have been detected in the studies conducted in the literature; however, the variability of the results among the studies might stem from the VEP measurement technique used in each study and the preferred settings in the measurement. Measurements are particularly affected by shape size and visual acuity. Retinal luminance and measurement field contrast are also important factors affecting results. We did not measure the pupil diameter affecting retinal luminance in our patients. And, since we were evaluating foveal vision, we used a small shape size. This makes the measurement most susceptible to visual acuity. Although we measured VEP with corrected refraction defects if the patients were wearing glasses, it is one of the points that can be discussed in our study.

Retinal dysfunction is confirmed by studies reporting impairment in PERG in PD, yet still, the disorder in the upper visual pathway cannot be excluded. Moreover, both PERG and VEP improve with treatment; however, there is an obvious difference: levodopa treatment improves PERG abnormalities to a higher degree than VEP deficiencies. One possible interpretation is that VEP changes in PD are secondary nondopaminergic and more chronic. Additional pathology beyond the retina appears to affect visual responses, including VEPs. Although the role of retinal dysfunction appears to be certain, the contribution of cortical and lateral geniculate disorder to these visual symptoms is unknown [41, 42].

Jindahra et al. noted a reduction in RNFL thickness in retrogeniculate lesions, pointing out that OCT measurements could represent both anterior and posterior pathways [43]. RNFL contains only unmyelinated axons from the retinal layers, RNFL thickness measurement may also be a way to monitor axonal loss in Parkinson's patients [30, 44, 45]. Thinning in peripapillary RNFL in PD was first shown in 2004 by Inzelberg et al. Peripapillary RNFL consists of ganglion cell axons. Although the thinning in RNFL is supported by many other studies, other studies reported different results [44, 45]. Inzelberg et al. found a significant decrease in RNFL thickness in the inferotemporal quadrant; Altıntaş et al. reported that the thinning began in the superior and inferior quadrants and that the temporal quadrant was affected in the advanced stages of the disease [20, 46]. In a study, measured outer retinal layers in neurodegenerative diseases were examined, and they found no significant difference [47].

If we look at retinal studies on the severity of PD disease; Lee et al. compared visual hallucinations and OCT findings in PD, and no significant relationship was found between retinal thickness and duration or severity of PD and drug dosages [48]. In the study by Kaur et al., no relationship was found between structural changes in the retina and the duration or severity of the disease. They emphasized that GCL-IPL thinning might be a more reliable parameter than RNFL thickness for the structural changes of the retina in PD [49]. In another study, it was emphasized that IRL that includes the nerve fiber layer, GCL, and inner plexiform layer thinning could be detected since most patients were at an early stage [50]. All amacrine cells, including dopamine-containing amacrine cells, are localized in the IRL layer close to ganglion cells. Evaluating OCT measurements in PD, the review article by Satue et al. pointed out that the IRL of the macular region are the most powerful biomarkers for the diagnosis and progression of PD [51].

Garcia–Martin et al. proposed foveal thickness to be the best parameter to predict more severe PD symptoms. They stated that OCT findings and PERG correlated with PD severity and Hoehn Yahr staging, yet no such result was obtained for VEP [52]. In another study, the OCT measurements in PD were evaluated with a new segmentation. They found that GCL can predict axonal damage in patients with PD. Segmentation analysis also revealed that the inner retinal layers of the macular region (RNFL, GCL, and IPL) were affected more by disease duration and GCL thickness was inversely proportional to disease duration and disease severity. Consequently, based on these recent segmentation studies, the IRL of the macular region have been shown as the strongest biomarkers in the diagnosis and progression of PH [53].

The decrease in macular volume in the retina was also found to correlate with the severity of the disease by Bodis Wollner et al. The macula is anatomically a region where ganglion cells are found more than a row thickness. RGC and RNFL are responsible for 30–35% of the thickness of the macula. Therefore, a decrease in macular volume and thickness can be expected in PD [20, 30]. Altıntaş et al. tested the relationship between UPDRS motor scores and retinal dopaminergic changes for the first time and found a significant correlation with loss of foveal thickness [20]. Huang et al. found morphological changes of retina in the PD patients with SD-OCT. They suggest that macular retinal thickness and macular volume decreasing could be detected in PD patients and macular volume may be more sensitive than other parameters in the retina. But these changes may not be apparent until the HY3 stage [54].

In another study, they suggest that OCT and OCT angiography (OCTA) combination can be a better diagnostic and progression marker of PD. Because of retinal vascular impairment of neurodegenerative diseases and great macular oxygen consumption of the retina [55]. Robbins et al. use OCT and OCTA in their study and they found that superficial capillary plexus vessel density and perfusion density decrease in PD compared with age-matched controls [56].

However, in the results of our study, we did not find a correlation between any of the OCT measurements or the VEP measurements and the severity of the disease. In our study, we evaluated the severity of the disease with UPDRS. We scored the UPDRS subsections separately and compared the total score separately. Since the modified Hoehn Yahr staging is already a staging that emphasizes the motor components, it has a strong correlation with the UPDRS total score and UPDRS part 3. However, this is expected. The second part of the UPDRS is a part that evaluates nonmotor characteristics and can be misleading due to the small number of questions and being answered by the patient and caregiver. However, for further studies, evaluation of the relationship between repeated measurements in OCT and disease severity and the response to treatment can provide valuable information in terms of disease progression.

Ocular imaging methods such as OCT enable the detection of axonal components of the anterior visual pathways, which are the direct part of the central nervous system related to vision. As the RNFL includes nonmyelinated axons, its measurement might be a way of monitoring axonal loss in Parkinson's patients. In studies conducted on MS patients, OCT measurement and RNFL thickness have been shown to correlate with brain volume. Studies on whether OCT measurements can functionally be a marker for disease progression in Parkinson's patients are needed.

The low number of patients in the control group and the fact that we did not use PERG, which provides functional retinal information, are some of the limitations of our study. PD prevalence also increases with age- and age-related retinal dysfunctions also do. We excluded the patients who have been diagnosed with retinal diseases from the study, so the lower patient number can be attributed to this. We did not measure the pupil diameter affecting retinal luminance in our patients and we use small shape sizes while doing VEP records. Although we corrected the refraction defect of the patients, these can affect the VEP results. VEP normal values can be prolonged with age and normal values of every laboratory can be different because of lots of environmental and technical issues. So we did not mention or discuss about the duration of our laboratory normal VEP values. Also, we did not assess the IPL layer, one of the OCT measurements, so this can be a limitation of our study. SD-OCT imaging speeds faster than TD-OCT and has good resolution. So this can minimize the artefacts caused by eye movements. But of course, there can be pitfalls and artefacts like defocusing, depolarization, and decentration. It is important to emphasize that; visual analysis of OCT image quality is affected by intraretinal abnormalities that are usually associated to particular ocular diseases, and opacities in the eye, operator-related factors. Optimal settings are necessary to avoid measurement errors [57]. Our operator is an ophthalmologist and visual acuity assessment, biomicroscopic anterior and posterior segment examination, applanation tonometry were done for every patient by her before OCT measurement. It is important to note that despite optimal conditions and exclusion of patients with ocular diseases that interfere with OCT measurement as described in the method section, there may still be artifacts in the measurement technique. Perhaps two consecutive but different measurements with two different ophthalmologists can minimize these artifacts. We performed the OCT measurement once in our study.

## 5. Conclusions

In this study, it was not confirmed that the retina can provide monitoring of the status of dopaminergic neurodegeneration and axonal loss in PD, although some studies have different findings from ours and the results of current studies are controversial. The use of OCTA in new studies may open new horizons in detecting noninvasive retinal changes in PD. So further studies are needed to determine a followup parameter for visual dysfunction that correlates with the clinical progression of the disease, with a large number of patients and control groups.

## Figures and Tables

**Table 1:** Demographic and clinical characteristics of the PD and the control groups.

	PD group	Control group	*p*-value
Age	65.58 ± 9.89	63.48 ± 11.23	0.411
Gender			
Women	14	17	0.053
Men	27	12	
Comorbidities			0.392
Diabetes mellitus (DM)	1		
Hypertension (HT)	4	2	
Coronary artery disease (CAD)	5	2	
Goiter	1	2	
Osteoporosis	3		
DM + HT	3	3	
DM + HT + CAD	2		
HT + COLD		1	
HT + CAD	3	1	
HT + goiter	2		
No comorbidity	17	18	

**Table 2:** LogMAR visual acuity values of PD and control groups.

Visual acuity	PD group		Control group	
	Right eye	Left eye	Right eye	Left eye
LogMAR 0, 0	12	8	7	5
LogMAR 0, 1	8	14	7	15
LogMAR 0, 2	16	14	14	8
LogMAR 0, 3	1	1		
LogMAR 0, 4	4	4	1	1

**Table 3:** OCT and VEP measurements between PD and control groups.

OCT and VEP localization	PD group	Control group	*p*-value
Right FT fovea	262 (219–319)	265 (229–327)	0.627
Right FT parafovea temporal	307 (248–356)	312 (257–355)	0.681
Right FT parafovea superior	317 (280–355)	319 (290–362)	0.960
Right FT parafovea nasal	320 (261–358)	319 (279–368)	0.837
Right FT parafovea inferior	314 (237–380)	314 (259–363)	0.876
Right FT perifovea temporal	289 (244–326)	283 (235–320)	0.559
Right FT perifovea superior	288 (262–320)	288 (258–312)	0.605
Right FT perifovea nasal	303 (266–345)	310 (274–344)	0.462
Right FT perifovea inferior	285 (249–342)	291 (233–309)	0.921
Right MV fovea	0.205 (0.172–0.251)	0.208 (0.180–0.257)	0.636
Right MV parafovea temporal	0.483 (0.389–0.558)	0.491 (0.403–0.557)	0.736
Right MV parafovea superior	0.501 (0.439–0.989)	0.501 (0.455–0.568)	0.857
Right MV parafovea nasal	0.503 (0.409–0.563)	0.501 (0.439–0.578)	0.837
Right MV parafovea inferior	0.494 (0.372–0.597)	0.494 (0.407–0.570)	0.866
Right MV perifovea temporal	0.906 (0.766–1.02)	0.890 (0.739–1.005)	0.580
Right MV perifovea superior	0.906 (0.824–1.814)	0.904 (0.812–0.995)	0.736
Right MV perifovea nasal	0.952 (0.835–1.083)	0.975 (0.860–1.082)	0.470
Right MV perifovea inferior	0.894 (0.783–1.073)	0.915 (0.732–0.970)	0.926
Right RNFL temporal	81 (58–111)	90 (63–142)	0.107
Right RNFL superior	133 (101–171)	127 (104–257)	0.499
Right RNFL nasal	70 (55–95)	80 (60–90)	0.119
Right RNFL inferior	134 (102–157)	135 (111–172)	0.686
Right GCC superior	96.57 ± 10.16	98.57 ± 7.45	0.384
Right GCC inferior	97.28 ± 10.11	99.92 ± 7.88	0.259
Right VEP amplitude	5.35 ± 2.09	5.47 ± 1.82	0.831
Right P100 latency	109.23 ± 6.92	108.37 ± 12.63	0.732
Right N75 latency	82.97 ± 11.48	81.12 ± 13.23	0.575
Right N135 latency	141.99 ± 9.65	143.87 ± 12.06	
Left FT fovea	259 (208–318)	263 (230–299)	0.600
Left FT parafovea temporal	311 (276–333)	312 (274–354)	0.943
Left FT parafovea superior	318 (235–352)	328 (276–359)	0.109
Left FT parafovea nasal	317 (223–353)	330 (297–366)	0.250
Left FT parafovea inferior	313 (236–339)	322 (283–359)	0.152
Left FT perifovea temporal	286 (244–308)	286 (270–313)	0.527
Left FT perifovea superior	286 (238–420)	296 (229–324)	0.340
Left FT perifovea nasal	306 (216–345)	309 (279–336)	0.128
Left FT perifovea inferior	285 (245–307)	292 (259–312)	0.539
Left MV fovea	0.203 (0.164–0.250)	0.206 (0.181–0.235)	0.579
Left MV parafovea temporal	0.439 (0.434–0.523)	0.490 (0.431–0.556)	0.929
Left MV parafovea superior	0.502 (0.369–0.946)	0.515 (0.434–0.563)	0.299
Left MV parafovea nasal	0.498 (0.350–0.554)	0.519 (0.467–0.574)	0.262
Left MV parafovea inferior	0.492 (0.371–0.532)	0.506 (0.444–0.565)	0.158
Left MV perifovea temporal	0.898 (0.767–0.968)	0.915 (0.807–0.984)	0.813
Left MV perifovea superior	0.900 (0.748–1.785)	0.931 (0.719–1.019)	0.493
Left MV perifovea nasal	0.963 (0.677–1.083)	0.971 (0.877–1.055)	0.140
Left MV perifovea inferior	0.896 (0.770–0.965)	0.917 (0.813–0.980)	0.535
Left RNFL temporal	81 (49–109)	81 (61–100)	0.939
Left RNFL superior	135 (99–183)	139 (102–182)	0.800
Left RNFL nasal	76 (52–103)	78 (60–118)	0.766
Left RNFL inferior	134 (98–184)	145 (121–155)	0.265
Left GCC superior	98.01 ± 10.27	99.95 ± 8.27	0.409
Left GCC inferior	99.13 ± 9.76	101.12 ± 7.23	0.361
Left VEP amplitude	5.19 ± 2.07	5.74 ± 2.15	0.330
Left P100 latency	109.30 ± 8.55	108.80 ± 10.51	0.843
Left N75 latency	82.67 ± 10.63	80.32 ± 12.52	0.442
Left N135 latency	145.15 ± 11.94	144.99 ± 13.72	0.960

MV: macular volume; FT: foveal thickness; RNFL: retinal nerve fiber layer; GCC: ganglion cell complex; VEP: visual evoked potentials. VEP latencies are given in milliseconds (ms) and amplitudes in microvolts (*μ*V). Variables distributed normally were expressed as mean ± standart deviation; variables distributed non-normally were expressed as median (minimum-maximum).

**Table 4:** VEP variables and UPDRS scores of the PD group.

	Patients number	Values
The duration of PD (yrs)	41	2 (0–18)
UPDRS1	41	4 (0–11)
UPDRS2	41	12.5 (3–37)
UPDRS3	41	19 (6–63)
UPDRS4	41	2 (0–11)
UPDRS total score	41	39 (14–109)
Right VEP P100 latency	40	109.23 ± 6.92
Left VEP P100 latency	41	109.3 ± 8.55
Right VEP amplitude	40	5.35 ± 2.09
Left VEP amplitude	41	5.19 ± 2.07
Right VEP N75	40	82.97 ± 11.48
Left VEP N75	41	82.67 ± 10.63
Right VEP N135	40	141.99 ± 9.65
Left VEP N135	41	145.15 ± 11.94

VEP latencies are given in milliseconds (ms) and amplitudes in microvolts (*μ*V). Variables distributed normally were expressed as mean ± standart deviation; variables distributed non-normally were expressed as median (minimum-maximum).

**Table 5:** Comparison between right and left eye VEP variables of PD group.

Student-T test for right and left VEP	
	*p*
Right-left VEP P100 latency	0.969
Right-left VEP amplitude	0.725
Right-left VEP N75 latency	0.895
Right-left VEP N135 latency	0.194

**Table 6:** *p*-values for the correlation between VEP variables and UPDRS score in the PD group.

Spearman correlation test	*p* values				
	UPDRS 1	UPDRS 2	UPDRS 3	UPDRS 4	UPDRS total
Right VEP P100 latency	0.753	0.646	0.767	0.691	0.795
Right VEP amplitude	0.787	0.170	0.677	0.897	0.426
Right VEP N75 latency	0.779	0.957	0.467	0.592	0.695
Right VEP N135 latency	0.262	0.529	0.476	0.152	0.355
Left VEP P100 latency	0.907	0.787	0.255	0.454	0.458
Left VEP amplitude	0.846	0.751	0.740	0.520	1.00
Left VEP N75 latency	0.779	0.957	0.467	0.592	0.645
Left VEP N135 latency	0.452	0.994	0.891	0.271	0.899

## Data Availability

The data used to support the findings of this study are available from the corresponding author upon request.
